# Key factors involved in reduction of damage to sunflower by the European sunflower moth in China through late planting

**DOI:** 10.1371/journal.pone.0250209

**Published:** 2021-04-22

**Authors:** Yunxia Cheng, Thomas W. Sappington, Lizhi Luo, Chenguang Liu, Yongjun Wang, Shuangping Liu, Zongze Zhang, Lijun Wang, Xingfu Jiang

**Affiliations:** 1 State Key Laboratory for Biology of Plant Diseases and Insect Pests, Institute of Plant Protection Chinese Academy of Agricultural Sciences, Beijing, China; 2 USDA-Agricultural Research Service, Corn Insects and Crop Genetics Research Unit, Ames, Iowa, United States of America; 3 Bayannur Station of Plant Protection and Quarantine, Bayannur, Inner Mongolia, China; 4 Fuzhou Customs District P.R. China, Fuzhou, Fujian, China; University of Carthage, TUNISIA

## Abstract

The European sunflower moth, *Homoesoma nebulellum* (Denis et Schiffermüller), emerged as a major new pest in Bayannur, China, in 2006. Insecticidal control with a single application is problematic because timing is critical, and multiple applications increase production and environmental costs. Management of *H*. *nebulellum* by planting date adjustment can be effective, but the optimal time window for late planting is unknown. Natural levels of *H*. *nebulellum* infestation were compared among sunflowers planted on five dates from April 25 to June 5 in two years, and the relationship between timing of adult abundance and flowering assessed. Delaying planting of sunflower from the traditional planting period of April 25 –May 5 to May 15 –June 5 significantly decreased damage by *H*. *nebulellum*. Seed infestation rate was 30–40 times higher, and number of larvae/head 75–100 times higher in the earliest two plantings than in the latest two. Within two years of implementing delayed planting in Bayannur city, infestation area decreased from 72% in 2006 to 1.5% in 2008, and production losses decreased from 4.5 ton/ha in 2006 to 0.36 ton/ha in 2008, a 97% decrease compared to 2006. Moreover, the infestation area caused by *H*. *nebulellum* was continuously controlled below 5.3% of the planting area since 2008. We found the overlap between the first two days of flowering and peak adult presence was the key factor influencing level of damage caused by *H*. *nebulellum*. Because the number of eggs laid in the first two days of flowering accounted for 68% of the total, and sunflower seed infestation rate was positively correlated with the number of trapped adults weighted by proportion of daily oviposition. Oviposition of the majority of eggs in the first two days of flowering suggests an evolutionary mechanism whereby females choose host plants most conducive to larval development, consistent with the preference-performance hypothesis.

## Introduction

The Bayannur city accounts for 28% of total sunflower, *Helianthus annuus*, production in China [1]. Emergence of the European sunflower moth, *Homoeosoma nebulellum* (Denis et Schiffermüller), in this city as a serious economic pest began in 2006 [2, 3], corresponding to greater areas of sunflower planting, new practices in cultivation, and the substitution of new cultivars for traditional ones. For example, in 2006, 70% of sunflower hectarage (94,000 ha) in Bayannur was damaged by *H*. *nebulellum*, causing economic losses of about 168 million dollars [2]. This insect also is an economic pest in other Eurasian countries [4, 5], such as Russia, France, Hungary, Spain and Turkey.

The life cycle and damage of the European sunflower moth is similar to that of the sunflower moth [6], *Homoeosoma electellum* (Hulst), which occurs mainly in the Americas, including the U.S., Canada, Mexico, and Cuba [7]. Female *H*. *nebulellum* prefer to deposit eggs on the inner wall of the early blooming disk flowers of the sunflower. First and second instars feed primarily on pollen [8]. Feeding by third instars may damage the stigmas and developing seeds, resulting in injured or empty seeds [9]. In addition, larvae lay a silk web over the surface of the disk flowers, thereby demarcating damage spots, which bind the dying parts of the florets with larval frass [10]. Accumulated debris in the damage spots predisposes the sunflower head to colonization by fungi like *Rhizopus*, and to bacterial infection [2]. Sunflower moth feeding and subsequent susceptibility to disease can severely decrease both the quantity and quality of seeds and oil production [2].

Minimizing production loss from *H*. *nebulellum* with insecticide is difficult, because treatment must target the first two instars to prevent the stigmas and developing seeds from damage [9, 11]. Susceptibility to control by chemicals is greatest in the first two instars because they feed on the surface of the disk, unlike older larvae which bore into the seeds where they are protected by the pericarp [9]. The period from first through second instar is about one week, and it is not easy for growers to time insecticide application to this stage for two main reasons. The first two instars of *H*. *nebulellum* are very small, averaging 2–3.6 mm long with a head capsule only 0.3–0.5 mm wide [9], making them difficult to detect. Likewise, infestation spots on the plant caused by young larvae are not apparent, especially during the early part of this period [2]. The larvae generally escape detection until the appearance of damage spots in the disks, by which time they have matured into older instars. If the early instar larvae are not detected and sprayed with insecticide in a timely manner, even increased rates of insecticide used later will be ineffective [12].

From 1985, when sunflower was first planted in Bayannur, until 2006 there were no outbreaks of *H*. *nebulellum*, which was virtually ignored by growers [11]. As a result, the pest’s biological characteristics, population fluctuation patterns and larval development in the disk were unfamiliar to them. For this reason, growers did not know the importance of proper timing of insecticide applications to target early-instar *H*. *nebulellum*. If this key stage is missed, keeping larvae under the economic injury level requires multiple applications of insecticide [13, 14]. Multiple applications of pesticides not only increases production costs, but also kills pollinators and disrupts natural ecological constraints on *H*. *nebulellum* populations by killing natural enemies [15]. Pollinators are important in production of sunflower, vegetables and fruits, which account for 15–30% of food produced for human consumption [16]. A pollinator deficiency decreases seed production, or increases production through the need for artificial pollination [17, 18]. In view of the above situation, there has been an urgent need to develop a new control strategy for *H*. *nebulellum* in Bayannur, preferably of high efficiency, low cost, ease of operation, and eco-friendliness.

Adjustment of planting dates has been used to control many pest insects and plant diseases. For instance, early planting of resistant varieties combined to reduce infestations of the Russian wheat aphid on barley in the western High Plains [19], while planting wheat 6 weeks later than the historical fly-free dates established before 1945 helps reduce infestations of Hessian fly [20]. Lint yield was greater for cotton planted in May than in June in Georgia due to reduced infestations of stink bugs [21]. Early sowing of green bean on July 3rd and variety selection showed potential to increase yield, improve quality and decrease disease incidence in Ethiopia [22]. Sowing date change has also been used in the control of other pests that damage sunflower. For example, late planting reduced damage ratings and percentage of damaged heads caused by seed maggot (*Neotephritis finalis*), sunflower bud moth (*Suleima helianthana*) [23], and banded sunflower moth (*Cochylis hospes*) [24]. Both early and late planting reduce infestations of sunflower moth *H*. *electellum* in Texas [13, 14, 25], and possibly in Kansas [26]. Whether adjustment of planting date can be used to control *H*. *nebulellum* in Bayannur was unknown.

Although the above studies on sunflower pests focused on efficacy of planting date as a pest control tactic, few examined the factors directly involved in determining the effectiveness of this strategy and how overlap of flowering and oviposition affects the degree of injury. In this study, we focused on two questions: 1) What time period for sunflower planting can be recommended so as to minimize economic losses from larval *H*. *nebulellum* while being agronomically acceptable in Bayannur? 2) What are the key factors affected by planting date that govern the extent of damage caused by *H*. *nebulellum* in sunflower?

## Materials and methods

### Planting date and infestation

Investigations were conducted in fields of cultivated sunflower in the Linhe District of Bayannur City, China. The experiment was designed as a completely randomized block with three replicates (plots) at each planting date in 2012 and 2013. Confectionery sunflower hybrid LD5009, the most popular variety in this area, was planted on five dates (April 25, May 5, May 15, May 25, and June 5) in fifteen plots in a whole field of more than 0.2 ha. Plot size was 10 m x 8 m. Wide (88 cm) and narrow (36 cm) row spacing alternated in each plot, with plastic membranes covering the surface of the soil between the narrow rows. The space between neighboring sunflower plants within a row was 30 cm, and there were 216 plants per plot. Plots were separated by a row of sunflowers.

When the plant advanced to the R7 stage (characterized by withering of ray flowers and the back of the disk turning pale yellow) [27], the head was cut and larvae counted from the seeds which were peeled off the disk. Fifty plants were randomly tagged in each plot before R7, during which all of them were used for estimating head infestation rate ($=\frac{number\;of\;infested\;heads}{number\;of\;heads\;for\;sample\;(50)}$). Ten plants in 2012, and 15 plants in 2013 were examined for larvae per head. The remaining heads were cut when the bracts became yellow and brown, indicating that plants were in the R9 stage and were physiologically mature [27]. Seeds harvested from these heads per plot were mixed and sun-cured for a month before they were weighed for plot yield. For each plot, five subsamples of >100 each of seeds were counted, weighed and averaged before the data were statistically analyzed for seed infestation rate ($=\frac{number\;of\;infested\;seeds}{number\;of\;seeds\;for\;each\;sample}$) and 100-seed weight ($=\frac{weight\;of\;each\;sample\times 100}{number\;of\;seeds\;for\;each\;sample}$).

### Sunflower planting area and infestation of *H*. *nebulellum* in Bayannur

Data of area infested and production loss caused by *H*. *nebulellum* in Bayannur from 1999 to 2020 was collected by the Bayannur Station of Plant Protection from each county.

### Adult monitoring

Adult *H*. *nebulellum* males were collected daily from May to September 2013 with traps baited with female sex pheromone (Pherobio Technology Co., Beijing). Traps consisted of a plastic basin (32cm diameter, 12cm depth) mounted 1.5m above the ground, filled to 80% of volume with 10% soapy water. Soap was added to reduce surface tension for collecting moths. Five traps were deployed in the cultivated field, of which four were baited with lure, and one with a blank lure (without lure and lure carrier over the basin) as a negative control. The lure was fixed with thin iron wire over the center of the basin and 3 cm above the water. Lures were changed monthly and water added weekly to account for evaporation. The traps were checked each morning, and captured moths were removed and counted.

### Monitoring of sunflower blooming period for each planting date

The first day of flowering was defined as the date when ray flowers became extended and 3–6 circles of disk flowers had blossomed. The end of flowering was recorded as the day when the last circle of disk flowers in the center had blossomed. The period from the first day to the end of flowering constitutes the R5 stage [27]. Sunflower blooming progress was monitored in 2013, and 20 plants before the R5 stage in each plot (totally 60 for each planting date) were tagged for sampling. From the first day of the R5 stage, the number of circles of blossoming disk flowers was recorded daily until all disk flowers withered. Blossoming in each circle of disk flowers usually lasted for 2–3 days. Typically, as two circles of disk flowers begin blossoming, disk flowers in adjacent outer circles wither. Thus, the number of circles of blossoming flowers on one head usually increases to a maximum and subsequently decreases to zero. The initiation date of flowering for a planting date was calculated as the mode value for the 60 plants observed. The daily number of blossoming circles of disk flowers for a planting date was calculated by summing that of the 60 plants on that day. Assuming disk flowers remained in bloom for two days, the period during which 50% or more of the plants were in the first or the second day of flowering was calculated and represented on flowering progress curves by a pair of vertical lines. If from the initiation day of flowering to the next day, the percentage of plants that were in the first two days of flowering remained below 50%, the period marked on the curve was extended to the next day, until the percentage exceeded 50%. The sampling plants were harvested at the R9 stage [27] and seed infestation rate per head was calculated and used for analyzing the relationship to adult number.

### Flowering and oviposition

In Bayannur, the flowering period or R5 stage lasts for seven to fourteen days for hybrid LD5009. Thus, the daily oviposition rate was measured during the first ten days of this period for the sunflowers planted on April 25, 2013. Fifteen heads were marked for each treatment day and for the control (day 0). Thus, a total of 165 heads were selected randomly before ray flowers extended, and a 40-mesh bag (25 cm × 25 cm) was tied over each to prevent oviposition by *H*. *nebulellum*. On each day of flowering, the bags were removed from the fifteen heads assigned for that treatment day for 24h, then re-bagged until cut. The controls were bagged throughout the experiment until cut (day 0). Beginning on the first day of flowering, the bagged heads were artificially pollinated twice at three-day intervals by using a dry sponge to transfer pollen from one head to another. All bagged heads were cut when the ray flowers withered, the back of the head had turned pale yellow and plants had advanced into the R7 stage [27]. The eggs were too small and concealed to be easily found in a flowering disk, hence we used the number of larvae instead. The number of larvae in a head, provided an index of oviposition on the day of flowering when that head was exposed.

### Relationship between population size of adult sunflower moths and rate of seed infestation

The relationship between the number of adult sunflower moths and seed infestation rate was explored in the data recorded in 2013. Twenty plants were marked in each plot (60 plants in all) for each planting date, to monitor sunflower blooming period and the correlated seed infestation rate. The number of males captured daily during the first 10 days of flowering (n1, n2, n3, …, n10), which was designated as the sunflower blooming period from the first day of disk opening to the tenth day, was used as an index of local adult population size under the assumption of a 1:1 sex ratio. Daily oviposition from the first to the tenth day (E1, E2, E3, …, E10) of flowering was used as a weighting factor; this allowed evaluation of oviposition preference in the flowering stage by calculating percentage of daily oviposition (p1, p2, p3, …, p10) as $pi\;=\frac{Ei}{\sum_{1}^{10}Ei}$. Adult number was weighted by number of eggs deposited from the first to the tenth day (Mean_1−10_) of flowering, which was calculated as ${\;Mean}_{1-10\;}\;=\sum_{1}^{10}ni\times pi$. For plants with the same flowering initiation date, these infestation rates were averaged before analyzing the relationship with adult number.

### Data analysis

The effect of the factor planting date on each dependent variable measuring infestation, which included head infestation rate, larvae/head, seed infestation rate, 100-seed weight, and plot yield, was analyzed by one-way ANOVA or a non-parametric test of independent samples. Before analysis, the data were tested for normality using the Shapiro-Wilk or Kolmogorov-Smirnov test. If the data were normally distributed, homogeneity of variances was tested further by using the Levene Statistic, followed by one-way ANOVA and Tukey’s HSD test to separate means. If the data were normally distributed but did not pass the homogeneity of variance test, they were analyzed by one-way ANOVA and Tamhane’s T2 test. If the data did not fit a normal distribution, they were analyzed using the non-parametric Kruskal-Wallis (*KW*) test with pairwise comparisons. The raw data for seed infestation rate and head infestation rate were arcsine transformed. Larvae per head and number of eggs among flowering days were transformed using a logarithmic function before testing for normality. When the raw number of larvae or eggs was 0, the corresponding logarithmic value was replaced with 0. The effect of planting date on these dependent variables was analyzed using the *KW* Test with pairwise comparisons. Larvae per head in 2013 was analyzed by one-way ANOVA and Tamhane’s T2 test. The effect of the factor planting date on the dependent variables of plot yield and 100-seed weight were analyzed by one-way ANOVA and Tukey’s HSD test. The relationship between adult number (weighted by the proportion of daily oviposition to total oviposition) as the independent variable and rate of seed infestation (dependent variable) was analyzed by both Pearson’s correlation and quadratic regression. In all cases, the significance threshold was α = 0.05.

## Results

### Planting date and infestation

The results of the two-year experiment showed that delaying planting of sunflower to the period from May 15 to June 5 significantly decreased damage by *H*. *nebulellum* compared to the traditional planting period from April 25 to May 5 (Table 1), as measured by head infestation rate (2012: *KW*_4, 10_ = 12.92, *P* = 0.012; 2013: *KW*_4, 10_ = 13.68, *P* = 0.008), larvae/head (2012: *KW*_4, 145_ = 104.04, *P* < 0.001; 2013: *F*_4, 220_ = 476.35, *P* < 0.0001, Tamhane’s T2 test), seed infestation rate (2012: *KW*_4, 10_ = 12.90, *P* = 0.012; 2013: *KW*_4, 10_ = 12.23, *P* = 0.016), 100-seed weight (2012: *F*_4, 10_ = 4.61, *P* = 0.02; 2013: *F*_4, 10_ = 10.34, *P* = 0.001), and plot yield (2012: *F*_4, 10_ = 10.06, *P* = 0.002; 2013: *F*_4, 10_ = 11.15, *P* = 0.001). Seed infestation rate of the early plantings on April 25 and May 5 was 30–40 times higher than that of the late plantings on May 25 and June 5, and larvae/head in the two early plantings was 75–100 times higher than that of the latter two plantings.

**Table 1:** Effects of sunflower planting date on measures of infestation by *H*. *nebulellum*.

Year	Planting date	Head infestation rate	Larvae/head	Seed infestation rate	100-seed weight (g)	Plot yield (kg)
2012	April 25	1.00±0.00a	105.10±38.15a	0.81±0.02a	10.95±1.28b	20.30±3.26c
	May 5	1.00±0.00a	31.80±10.11a	0.65±0.01ab	11.34±0.67ab	23.00±3.02bc
	May 15	1.00±0.00a	12.00±2.00b	0.46±0.01ab	13.29±0.32ab	32.73±1.01ab
	May 25	0.49±0.11b	1.73±0.22b	0.02±0.01b	14.02±0.59ab	38.32±0.92a
	June 5	0.47±0.11b	1.70±0.67b	0.02±0.01b	14.43±0.36a	35.57±3.08a
2013	April 25	1.00±0.00a	85.22±8.72a	0.67±0.05a	9.13±1.15c	25.29±6.64bc
	May 5	1.00±0.00a	72.22±5.87a	0.60±0.04a	9.69±0.46bc	20.31±3.12c
	May 15	0.94±0.03ab	14.78±1.53b	0.34±0.03b	12.22±0.39ab	34.98±4.63bc
	May 25	0.23±0.04b	0.96±0.28c	0.02±0.02c	13.39±0.54a	40.62±2.32ab
	June 5	0.46±0.01ab	0.84±0.24c	0.02±0.01c	13.56±0.30a	54.90±1.46a

Means ± SEM in each column within year followed by the same letter are not significantly different (α = 0.05) by Tukey’s HSD test, Tamhane’s T2 test, or Kruskal-Wallis test. Sample size was three, except for larvae/head where n = 30 in 2012 and n = 45 in 2013.

### Delayed planting for management of sunflower moth in Bayannur

**Fig 1:**
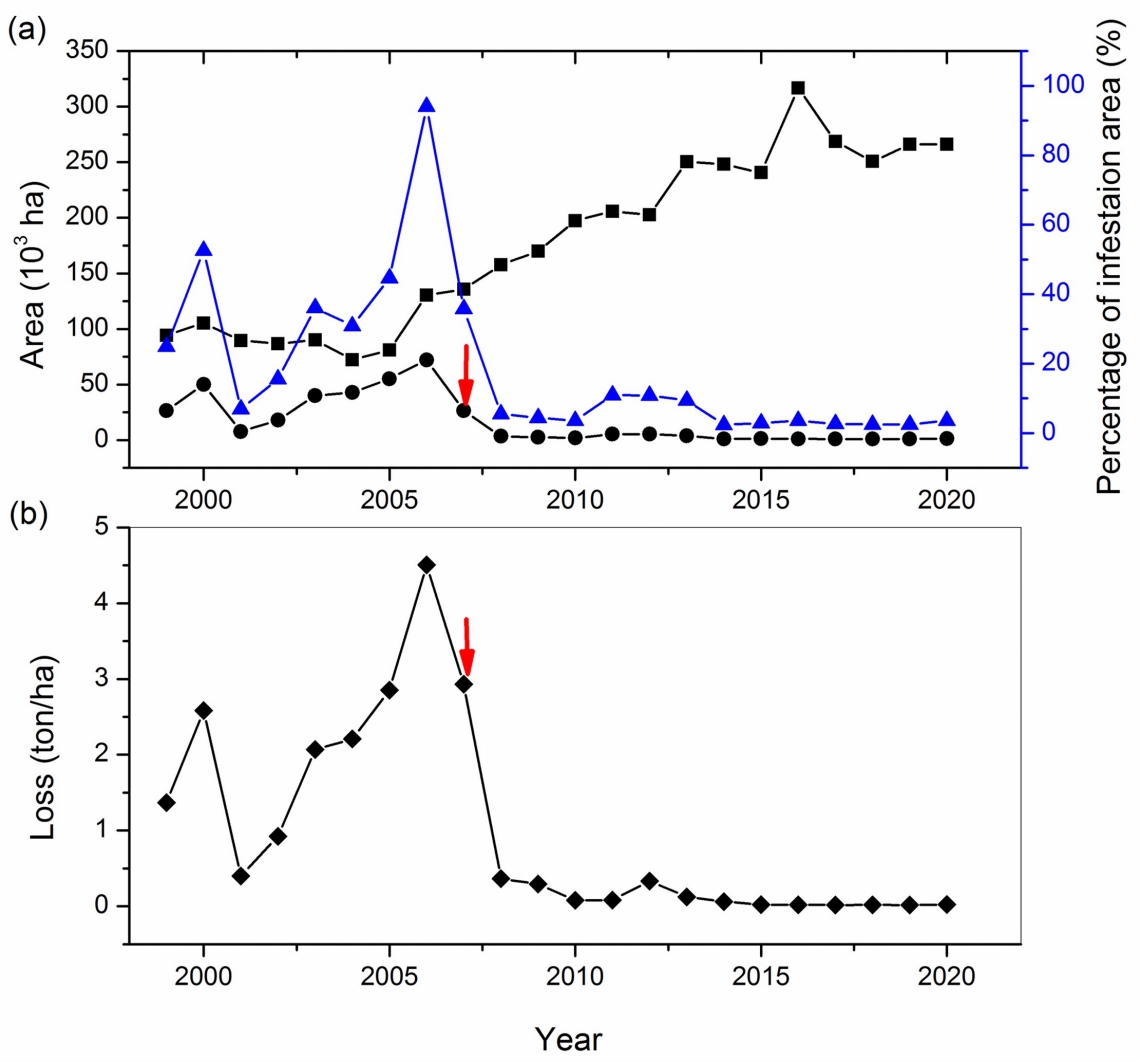
Area planted to sunflower and area of infestation of sunflower by *H*. *nebulellum* in Bayannur from 1999 to 2020. (a) Area of sunflower planting and infestation, and (b) sunflower production loss. (a) The symbol square, circle, and up triangle indicates planting area, infestation area, and percentage of infestation area, respectively. Red arrows indicate first partial regional implementation of delayed planting dates in 2007. Data was from Bayannur Station of Plant Protection.

When delayed planting was first implemented on a large commercial scale in Bayannur, hectarage infested with *H*. *nebulellum* decreased from 72% in 2006 to 26% in 2007 (Fig 1A). With increased adoption of delayed planting in 2008, the area of infested sunflowers decreased further to 3.5%. Sunflower infestation area was continuous controlled below 5.3% of the planting area from 2008 to 2020, even as total sunflower hectarage increased (Fig 1A). Production loss decreased from 4.5 ton/ha in 2006 to 2.9 ton/ha in 2007, and it continuously decreased to 0.36 ton/ha in 2008, a 97% decrease compared to 2006 (Fig 1B). Both the infestation area and production loss since 2008 generally have been less than before the outbreak of *H*. *nebulellum* in 2006, and farmers have not reverted to use of insecticides to prevent damage by *H*. *nebulellum* since implementation of delayed planting.

### Flowering and adult occurrence

**Fig 2:**
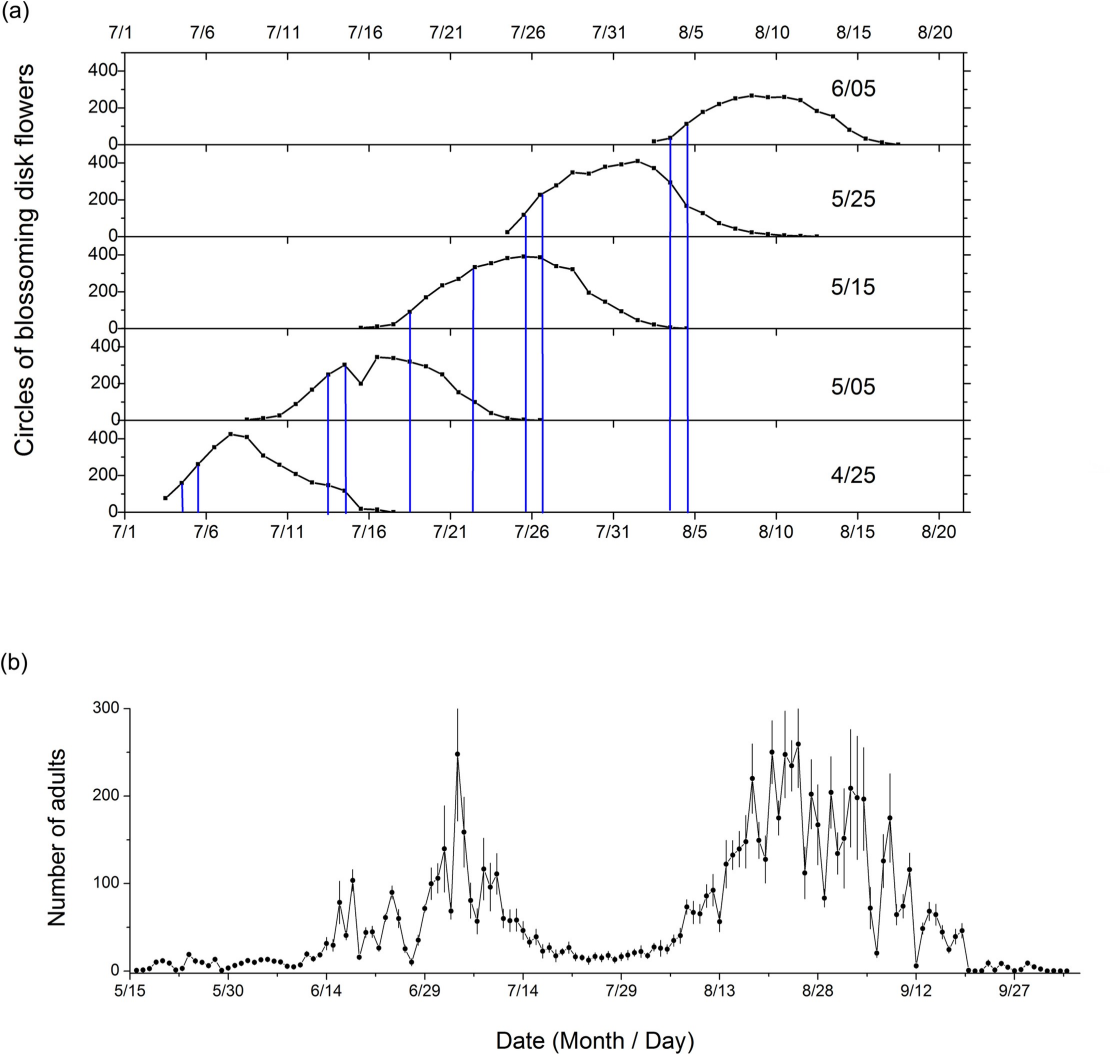
Adult *H*. *nebulellum* occurrence measured by pheromone trapping, and flowering of sunflower in Bayannur, China, in 2013. (a) Temporal overlap between flowering period of sunflowers planted on different dates and occurrence peak of adult *H*. *nebulellum*. Blue lines pairs demarcate day from first flowering to the day when 50% of plants entered the first or second day of flowering for each planting date group. The number of circles of blossoming disk flowers was the sum of 60 sampled plants. (b) Mean (± SEM, n = 4) number of adult male *H*. *nebulellum* captured by pheromone traps.

There were two generations of *H*. *nebulellum* in Bayannur, with trap captures of adults typically peaking from June 15 to July 15, and from August 10 to September 20 (Fig 2B). The full bloom stage of sunflower was identified as the period during which 50% or more of the plants entered the first or the second day of flowering. The full bloom stage of sunflower sown at the two earliest dates tested began on July 5, and July 14 (Fig 2A), which coincided with the period of the first adult flight (Fig 2B). Such high coincident timing between flowering period and peak adult moth activity may expose both head and seed to severe infestation. By contrast, when sowing date was delayed to May 25 or June 5, the beginning of full bloom stage was delayed to July 26, and August 4, respectively (Fig 2A). This, coincided with the intervening period between the two peaks of adult activity (Fig 2B), which protected sunflower from being damaged by *H*. *nebulellum*. However, delaying planting date for another 10–20 days after June 5 would synchronize the blooming stage with the second adult flight, and thus expose the crop to a late infestation of *H*. *nebulellum*.

### Effect of flowering period on oviposition

**Fig 3:**
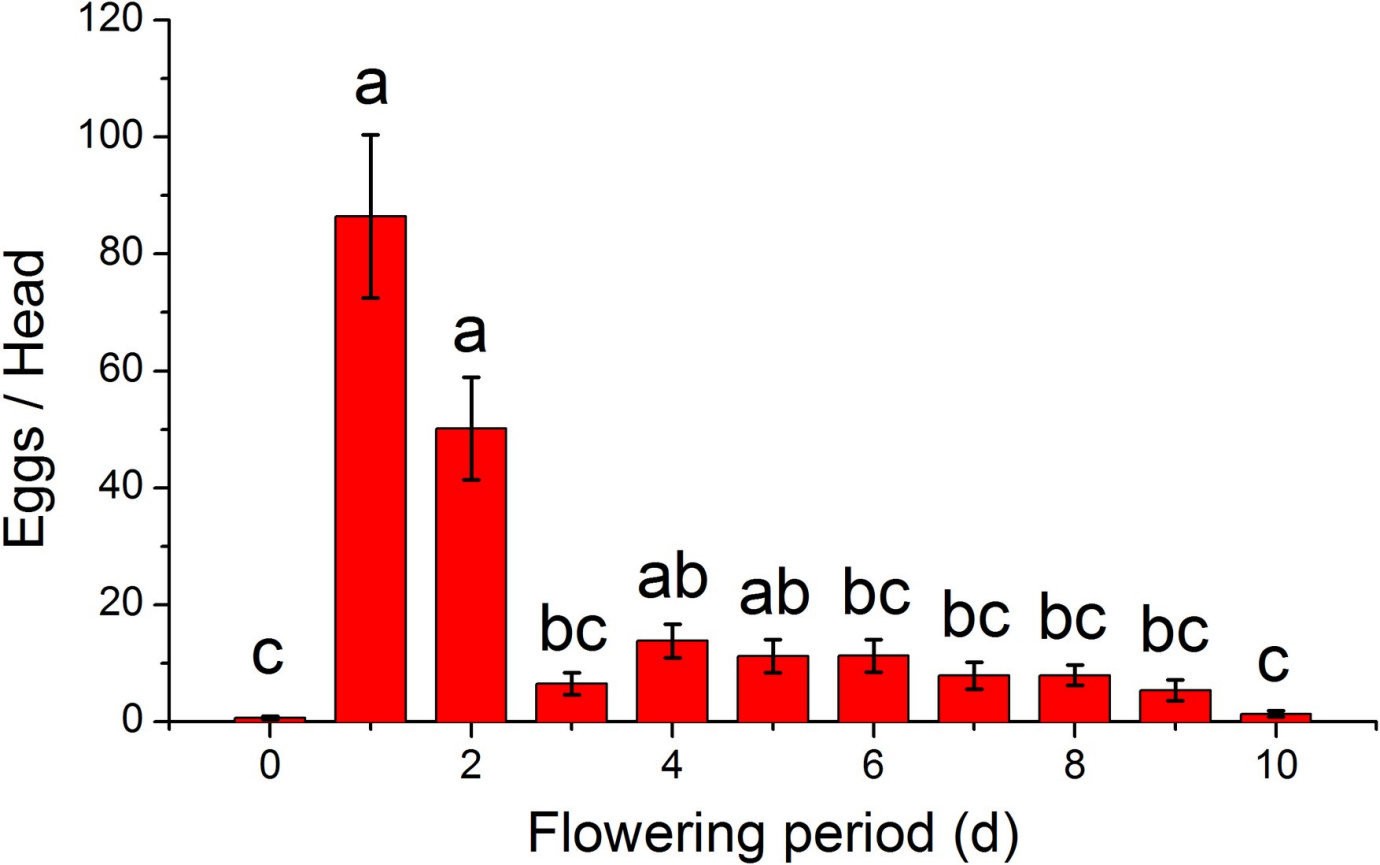
Daily oviposition of *H*. *nebulellum* on sunflower head across period of flowering. Day 0 indicates negative control, in which flower heads were bagged across the full testing period. Means (± SEM) sharing the same letter are not significantly different (α = 0.05) analyzed by Kruskal-Wallis test. Sample size per group was 15.

Daily oviposition of *H*. *nebulellum* was affected significantly by the flowering period of sunflower (Fig 3). We found that *H*. *nebulellum* tends to oviposit most during the first two days of flowering (*KW*_10, 164_ = 93.69, *P* < 0.0001), which accounted for 68% of the total oviposition measured over the first 10 days of flowering.

### The relationship between adult number and seed infestation rate

**Fig 4:**
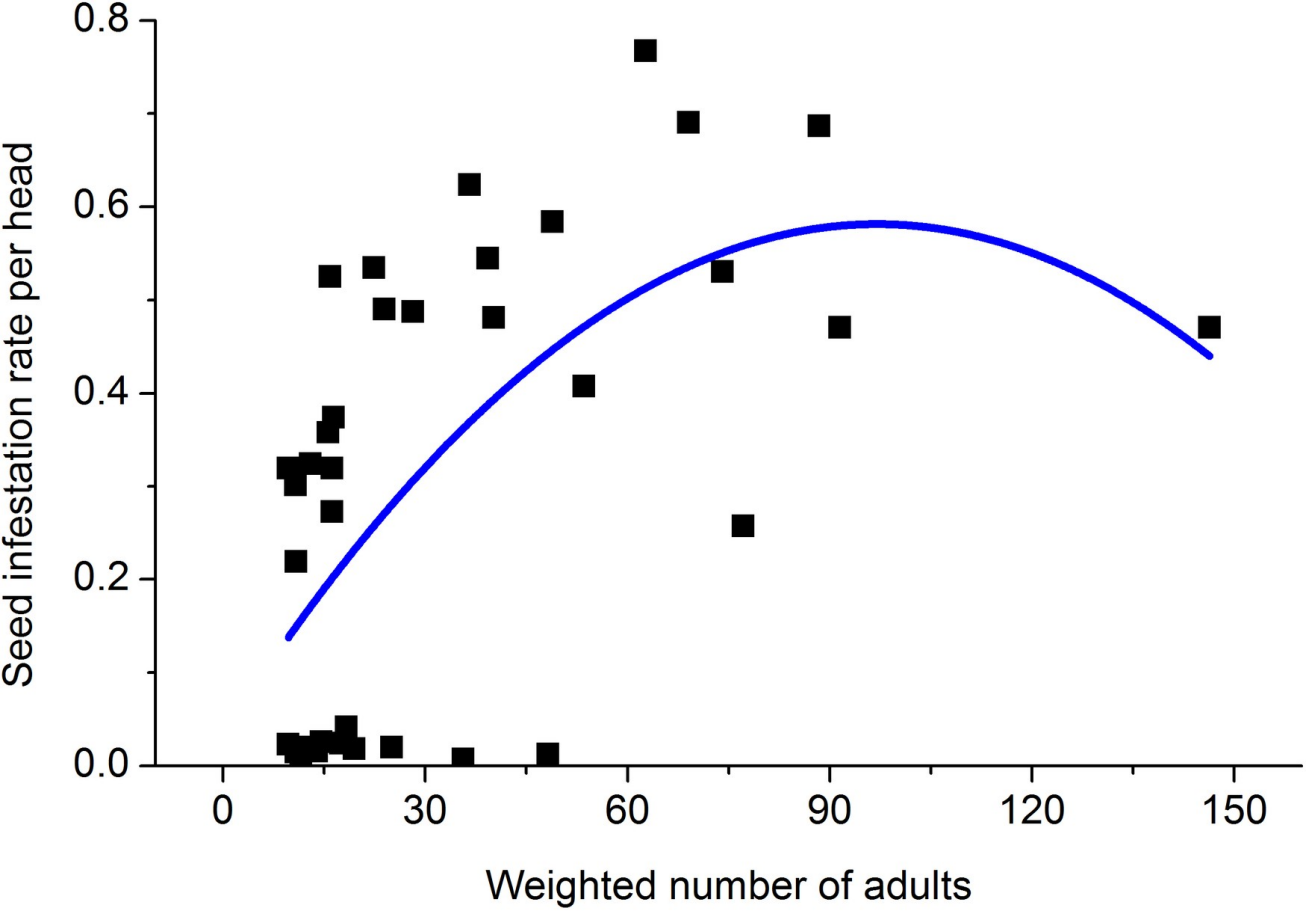
Quadratic regression of seed infestation rate per sunflower head on number of *H*. *nebulellum* adults weighted by proportion of daily oviposition to total oviposition. The sample size was 38.

The synchrony between flowering and adult occurrence peaks can be represented numerically as the average number of adults from the first day of flowering to the tenth day, weighted by daily oviposition expressed as a proportion of total oviposition. The relationship between *H*. *nebulellum* damage and sunflower flowering is reflected by the relationship between weighted adult number and rate of seed infestation, which is described fairly well by a quadratic curve (Fig 4; y = 0.03+0.01x-5.82x^2^, *p* < 0.0001, *r* ^2^ = 0.37). Adult number during the first two days of flowering contributed more to the infestation of seeds on a single disk than during the remaining period of flowering (Pearson’s correlation analyses, day 1–2: *r* = 0.52, *p* = 0.001, n = 38; day 3–10: *r* = -0.007, *p* = 0.97, n = 38).

## Discussion

### Planting date and infestation

The damage caused by *H*. *nebulellum* decreased with the delay of planting from April 25 to June 5 in both years of experiments in Bayannur. Both seed infestation rate and disk infestation rate decreased with the delay, while plot yield and 100-seed weight increased. The infestation level was significantly less when planting on May 25 and June 5 than on the three earlier plantings of April 25, May 5, and May 15 in the two years of experiments. Accordingly, we suggest that the best planting date for sunflower in Bayannur to reduce damage from sunflower moths, is from May 25 to June 5, rather than the traditional planting time from late April to mid-May. This new planting period recommendation was suggested to the sunflower industry and has been adopted since it was first suggested in 2007 in Bayannur. This has decreased the proportion of sunflower area infested from 72% in 2006 to 1.3% in 2020. Concomitantly, production loss has decreased from 4.5 ton/ha in 2006 to 0.02 ton/ha in 2020 (Fig 1B). Delayed planting has prevented huge economic losses from *H*. *nebulellum* infestation for local farmers whose livelihoods depend on growing sunflower. Before the use of delayed planting, *H*. *nebulellum* was controlled with frequent insecticide applications, costing farmers $75–150 per hectare annually [9]. At least $15 million has been saved annually by reducing infestations of sunflower moth via delayed planting in Bayannur [11].

Although delayed planting can decrease infestations by *H*. *nebulellum*, delaying to a date later than June 5 may have adverse effects on sunflower production. When planting was delayed to June 5, the physiological maturation of sunflower was delayed to around September 20, which was close to the limit of maturation time in this area. According to historical meteorological data for Bayannur, minimum temperature falls to below 0°C in October (China National Meteorological Science Data Center). So delaying the planting date too long increases the risk of encountering frost before physiological maturation. Furthermore, delaying planting until June may decrease seed yield and oil content [28]. In addition, the quantity of empty grain in the May 25 and June 5 plantings increased more than two-fold compared to those planted on April 25 and May 5 (S1 Table), suggesting that decrease in pollinator abundance later in the season may limit how long planting can be delayed. Early planting has also been reported to be effective in insect management. For example, early planting decreased seed damage caused by red sunflower seed weevil, *Smicronyx fulvus*, in North Dakota and Nebraska [29]. The mechanisms involved in the effect of planting date adjustment on pest control warrant further study.

### Key factors involved in the later planting

Our results indicate that a period of low oviposition between the two adult generations of *H*. *nebulellum* in Bayannur each summer, provides sunflower a potential window of escape from attack during flowering. The recommended planting date from May 15 to June 5 ensures the flowering period coincides with this period of low adult population density. We found that female *H*. *nebulellum* prefer to oviposit on the disk during the first two days of flowering, accounting for almost 70% of a female’s total fecundity, which is the key factor need to be considered in the later planting of sunflower to control the infestation of *H*. *nebulellum*. In other words, the more plants avoid the overlapping between the first two days of flowering and adult occurrence peak, the less plants will be infested by larval *H*. *nebulellum* theoretically. In contrast, *H*. *electellum* females oviposit over a relatively extended period, with about 70% of a female’s eggs being laid during the first six days after the heads have opened [13]. Nevertheless, these findings indicate that both *H*. *nebulellum* and *H*. *electellum* females prefer the early stage of blossoming disks for oviposition. Our results are supported by laboratory studies by Metayer *et al*. (1991) [4] who found *H*. *nebulellum* females laid most of their lifetime eggs during the first two days of oviposition.

Pollen is an essential oviposition stimulant for female *H*. *electellum* [30]. However, fresh pollen is released daily for almost 10 days from the commencement of flowering, and cannot explain why adults preferentially oviposit in disks during the first two days of flowering. Under the preference-performance hypothesis, a female should prefer to oviposit on the plants most likely to be suitable for successful development of its offspring [7, 31]. This hypothesis is supported by a study of *H*. *electellum*, in which female oviposition preference was positively correlated with larval growth performance on sunflower pre-breeding lines [32]. In the case of *H*. *nebulellum*, the observed strong oviposition preference for sunflowers in the first two days of flowering may provide a female’s offspring maximum time for utilization of optimal neonate food, i.e. the blooming disk flowers. Because it takes 2–3 days for the eggs to hatch in July in Bayannur [2], neonate feeding will occur during the middle to late period of flowering on a disk. It takes about 3–4 days to advance through the neonate stage (instars 1–2), the most precarious stage for the larvae, during which they must feed on pollen to garner the energy and nutrients necessary for survival [7]. By the time the larvae pass the neonate stage and no longer need to feed on flowers, most of the disk flowers have withered. Adult oviposition preference in the banded sunflower moth, *Cochylis hospes*, is stimulated in part by plant volatiles from the sunflower head [33]. Given the preference for oviposition in the first two days, we suspect that ray and disk flowers may play different roles in stimulating oviposition behavior of *H*. *nebulellum*. Ray flowers may tend to attract females for oviposition with their distinctive volatiles, while disk flowers serve to feed the resulting larvae throughout blooming, circle by circle.

### Relationship between adult occurrence and the larval damage

Several studies have inferred that flowering may be linked to damage of *H*. *nebulellum* or *H*. *electellum* [13, 34]. But until now, how flowering of the sunflower head is related to damage by larvae was unclear. In this study, we clarified that 1) flowering period, determined mainly by planting date, affected larval infestation; and 2) synchrony between flowering and adult occurrence peaks was positively correlated with the seed infestation rate, which is well described by a quadratic curve (Fig 4). The first two days of flowering accounted for almost 70% of adult numbers. We conclude that the most important factor contributing to the success of the delayed planting strategy in minimizing *H*. *nebulellum* injury to sunflower is desynchronizing the flowering period, especially the first two days, and seasonal flight peaks. This is the first report clarifying the relationship between flowering of sunflower and *H*. *nebulellum* infestation.

## Supporting information

S1 Table Effect of planting date on rate of empty grains of sunflower. Data was collected from the same plots in the experiment of 2013. There are three samples for each planting date, and one hundred seeds for each sample. Columns sharing the same letter are not statistically different (one-way ANOVA and Tukey’s HSD test).
(DOCX)

S1 Data (XLSX)
