# Supplementary material for: Key factors involved in reduction of damage to sunflower by the European sunflower moth in China through late planting
Source: PLoS One. 2021 Apr 22;16(4):e0250209. doi: 10.1371/journal.pone.0250209 (PMC8061923; doi:10.1371/journal.pone.0250209)
Supplement: S1 Table — Data was collected from the same plots in the experiment of 2013. There are three samples for each planting date, and one hundred seeds for each sample. Columns sharing the same letter are not statistically different (one-way ANOVA and Tukey’s HSD test). (DOCX) [file pone.0250209.s001.docx]

# S1 Table.

## Effect of planting date on rate of empty grain

Rate of empty grain, a condition indicating lack of pollination, which was recorded in 2013, was affected significantly by planting date (*F* _4, 10_ = 8.41, *P* = 0.003). Rates of empty grain for the two latest plating dates are almost two-fold greater than for the two earliest planting dates (S1 Table, *P* ≤ 0.019).

S1 Table. Effect of planting date on rate of empty grains of sunflower.

| Planting date | Rate of empty grains (%) |
| --- | --- |
| 25 April | 5.94±1.83b |
| 5 May | 5.87±0.87b |
| 15May | 9.07±0.86ab |
| 25 May | 13.74±1.95a |
| 5 June | 13.47±0.53a |

Data was collected from the same plots in the experiment of 2013. There are three samples for each planting date, and one hundred seeds for each sample. Columns sharing the same letter are not statistically different (one-way ANOVA and Tukey’s HSD test).
